# Differential Effects of Allergen Challenge on Large and Small Airway Reactivity in Mice

**DOI:** 10.1371/journal.pone.0074101

**Published:** 2013-09-06

**Authors:** Chantal Donovan, Simon G. Royce, James Esposito, Jenny Tran, Zaridatul Aini Ibrahim, Mimi L. K. Tang, Simon Bailey, Jane E. Bourke

**Affiliations:** 1 Lung Health Research Centre, Department of Pharmacology & Therapeutics, University of Melbourne, Parkville, Victoria, Australia; 2 Department of Pediatrics, University of Melbourne, Parkville, Victoria, Australia; 3 Department of Allergy & Immunology, Murdoch Children’s Research Institute, Royal Children’s Hospital, Parkville, Victoria, Australia; 4 Faculty of Veterinary Science, University of Melbourne, Parkville, Victoria, Australia; Research Center Borstel, Germany

## Abstract

The relative contributions of large and small airways to hyperresponsiveness in asthma have yet to be fully assessed. This study used a mouse model of chronic allergic airways disease to induce inflammation and remodelling and determine whether *in vivo* hyperresponsiveness to methacholine is consistent with *in vitro* reactivity of trachea and small airways. Balb/C mice were sensitised (days 0, 14) and challenged (3 times/week, 6 weeks) with ovalbumin. Airway reactivity was compared with saline-challenged controls *in vivo* assessing whole lung resistance, and *in vitro* measuring the force of tracheal contraction and the magnitude/rate of small airway narrowing within lung slices. Increased airway inflammation, epithelial remodelling and fibrosis were evident following allergen challenge. *In vivo* hyperresponsiveness to methacholine was maintained in isolated trachea. In contrast, methacholine induced slower narrowing, with reduced potency in small airways compared to controls. *In vitro* incubation with IL-1/TNFα did not alter reactivity. The hyporesponsiveness to methacholine in small airways within lung slices following chronic ovalbumin challenge was unexpected, given hyperresponsiveness to the same agonist both *in vivo* and *in vitro* in tracheal preparations. This finding may reflect the altered interactions of small airways with surrounding parenchymal tissue after allergen challenge to oppose airway narrowing and closure.

## Introduction

Asthma is characterized by inflammation, airway wall remodelling and airway hyperresponsiveness (AHR), whereby airways are more sensitive to a variety of stimuli and subsequently contract too easily and too much [1]. The relative contributions of changes in airway smooth muscle (ASM) function and of the influence of the altered airway environment to AHR remain unclear.

There has been increased interest in assessing the role of small airways (defined as <2 mm diameter in adults) in asthma [2], [3]. Small airway resistance is significantly higher in mild asthmatics than healthy subjects [4], [5], associated with increased inflammation and remodelling in the distal lung [3]. *In vitro* studies performed under isometric conditions have also shown that small airways have relatively increased sensitivity to contractile mediators than larger airways [6], suggesting that they may contribute significantly to AHR.

Various animal models of allergic airway disease (AAD) have been used to elucidate mechanisms underlying the development of AHR following chronic allergen challenge [7]. The majority of these studies have demonstrated AHR by assessing whole lung responses *in vivo* and/or the development of force in conveniently accessible large airways in organ bath studies under isometric conditions *in vitro* [8], [9], [10]. The use of lung slices, in which intact small airways maintain cell-cell and cell-matrix interactions with surrounding parenchyma, provides a physiologically relevant *in vitro* setting in which to explore the influence of allergen on small airway reactivity [11], [12], [13], [14]. Using this approach, a single study using a chronic ovalbumin (OVA) challenge model has reported that small airway responsiveness to a single maximally effective concentration of acetylcholine (ACh) was unchanged, despite airway remodelling and *in vivo* AHR to methacholine (MCh) [14]. However, a potential limitation of this study was the failure to assess whether a sustained inflammatory environment is required to sustain hyperresponsiveness. This may require *ex vivo* incubation of lung slices with inflammatory cytokines such as interleukin-1 (IL-1) and tumour necrosis factor α (TNFα), which have previously been demonstrated to induce AHR in tracheal preparations *in vitro* [15], [16].

The current study extends these limited findings, using a mouse model of AAD in which *in vivo* AHR is induced by chronic challenge with low levels of aerosolized OVA for >6 weeks. Other key features include airway inflammation and remodelling, characterized by epithelial hyperplasia and subepithelial fibrosis without thickening of the airway smooth muscle layer [17]. Measurements of *in vitro* force development in large airways and changes in small airway lumen area were made to a full range of MCh concentrations in this model to provide insights into their relative contributions to confirmed *in vivo* AHR.

## Methods

### Ethics Statement

The experimental procedures described in this manuscript were approved by Animal Experimentation Ethics Committees of the University of Melbourne (approval #1011608) and Murdoch Children’s Research Institute (MCRI, approval # A652) and conducted in compliance with the guidelines of the National Health and Medical Research Council (NHMRC) of Australia on animal experimentation.

### Chronic Ovalbumin Challenge Model of Allergic Airway Disease

Female Balb/C mice (6–12 weeks) were subjected to a chronic model of AAD as described previously [17]. Briefly, mice were administered grade V chicken egg OVA (10 µg OVA per 0.4 mg aluminum potassium sulfate adjuvant (alum) in 0.5 mL saline i.p.) on days 0 and 14 and nebulised OVA (2.5% w.v^−1^ saline) three times per week between days 21–63. Control mice received equivalent volumes of adjuvant and nebulised saline over the same period. Measurements of *in vivo* reactivity and subsequent collection of blood and bronchoalveolar lavage (BAL) fluid occurred on day 64 or 65. As detailed later, tissues for *in vitro* reactivity studies were collected from naïve mice or from separate mice after the same challenge protocol to avoid any influence of tracheal cannulation or drug treatment *in vivo* on subsequent airway responses.

### Measurement of Airway Reactivity *in vivo*

Whole lung reactivity was assessed by direct plethysmography (Buxco Electronics, Troy, NY). Mice were anaesthetised with i.p. ketamine/xylazine (200 µg.g^−1^ and 10 µg.g^−1^ respectively) prior to tracheotomy and jugular vein cannulation. Mice were ventilated with a small animal respirator (Harvard Apparatus, Holliston, MA) delivering 0.01 ml.g^−1^ bodyweight at a rate of 120 strokes.min^−1^. Airway resistance was measured (Biosystem XA; Buxco Electronics) for 2 min after each increasing intravenous MCh dose.

### Determination of OVA-specific IgE Levels

Serum obtained by lethal cardiac puncture of anaesthetised mice was stored at −70°C for ELISA of OVA-specific IgE [18], with levels expressed as units relative to a standard curve generated from positive-control sera. OVA-specific IgE was only detected after allergen challenge (38.5±13.0 units, n = 6).

### Bronchoalveolar Lavage

After cardiac puncture, BAL was performed by infusion and extraction of 3×1 ml of ice-cold saline (pooled mean volume, 2.5±0.3 ml). Total viable cellularity was measured in a haemocytometer using trypan blue exclusion. Differential cell counts were determined in counts of 300 cells from cytospin smears of 4×10^5^ BAL cells stained with Diff-Quick (Life Technologies, Auckland, New Zealand).

### Lung Histology

Right lung lobes fixed in 10% neutral buffered formalin for 18–24 h were routinely processed. Serial 5 µm sections were assessed for subepithelial collagen deposition and goblet cell metaplasia by staining with Masson trichrome and Alcian blue-periodic acid Schiff (AB-PAS) respectively.

### Measurement of Airway Reactivity *in vitro* - Trachea

Tracheal segments, approximately 2 mm in diameter, were collected from saline- or OVA-challenged mice on day 64 or 65, or from naïve mice and mounted on the same day in temperature-controlled baths of a Mulvaney-Haplern wire myograph (Danish MyoTechnology; Aarhus, Denmark) containing 5 ml Krebs-Henseleit buffer solution (59 mM NaCl, 2.3 mM KCl, 0.69 mM MgSO_4_. 7 H_2_O, 2.5 mM CaCl_2_.6 H_2_O, 0.6 mM KH_2_PO_4_, 10 mM EDTA, 25 mM NaHCO_3_ and 6 mM glucose; pH 7.4, 37°C, aerated with 95% O_2_/5% CO_2_). Wires threaded through the airway lumen were attached to a micrometer and to a force displacement transducer to record changes in isometric tension (ΔmN) using Power Lab and Chart software (ADI Instruments). An optimal resting tension of 1.5–1.8 mN was determined for each tissue based on the maximum response to Depolarising Potassium Solution (DKS: containing 123.7 mM K^+^), prior to generation of cumulative concentration-response curves to MCh.

### Measurement of Airway Reactivity *in vitro* – Small Airways

#### Preparation of lung slices

Lung slices were prepared as previously described [19]. Briefly, lungs were inflated with warm 2% w/v ultra pure low melting point agarose (Invitrogen) in Hank’s Balanced Salt Solution supplemented with 40 mM HEPES (sHBSS) via a tracheal cannula, followed by a small bolus of air to push the agarose into alveolar spaces. The agarose was solidified at 4°C for 20 min, then a single lobe was mounted in a vibratome in cold HBSS (VT 1000S, Leica Microsystems) to prepare serially sectioned 150 µm slices cut at a similar distance from the lung periphery in both groups.

Since it is not possible to define the airways within lung slices in terms of airway generation relative to the trachea, we used airways previously described as 3–4 generations higher than the most distal airways with an intact epithelium containing ciliated cells as small airways [20]. These airways have been shown to have greater reactivity to MCh than more proximal or distal airways [20]. Consistent with previously reported remodeling changes in this model [17], airway lumen diameter in these slices was not affected by OVA challenge. This removed airway size as a confounding variable that could contribute to altered reactivity.

#### Lung slice culture and cytokine incubation

Prior to assessment of small airway reactivity, slices were cultured in 24 well plates for up to 72 h in Dulbecco’s Modified Eagle’s Medium (DMEM) supplemented with 1% penicillin-streptomycin solution, at 37°C and 5% CO_2_. Some slices were incubated for 48 h in the presence of inflammatory cytokines IL-1α (10 ng.mL^−1^, Sigma) and/or TNFα (50 ng.mL^−1^, R & D systems). These cytokine concentrations have been previously shown to induce tracheal AHR *in vitro* [15], [16].

#### Acquisition of small airway images

Phase contrast microscopy was conducted using an inverted microscope (Eclipse Ti-U; Nikon) using 10×objective lens, zoom adaptor, reducing lens and camera (CCD camera model TM-62EX; Pulnix). Individual slices were placed in a custom-made perfusion chamber between 2 coverglasses (approximately 100 µL volume) and covered in fine wire mesh (Small Parts Inc.) with a small hole cut over the airway under examination. A single airway (150–400 µm) was selected for experimentation based on the presence of an intact layer of epithelial cells displaying ciliary activity.

#### Measurement of responses to methacholine

Using a gravity-fed system, lung slices were perfused at a constant rate for each condition. Some slices were permeabilized to Ca^2+^ by simultaneous treatment with 20 mM caffeine and 50 µM ryanodine. This pharmacological approach permits the assessment of MCh responses due to RhoA/Rho kinase-mediated Ca^2+^ sensitisation alone. Full details have been reported previously [21].

#### Image analysis

Digital images (744×572 pixels) were recorded in time lapse (0.5 Hz) using image acquisition software (Video Savant; IO industries, Inc.), converted to a TIFF file and analysed using PC software NIH/Scion (Scion Corporation; download www.scioncorp.com). An appropriate grey scale threshold was chosen to distinguish between the airway lumen and surrounding tissue, with lumen area in each image calculated by pixel summation.

### Statistical Analysis

*In vivo* and tracheal responses to MCh were expressed as increases in resistance (ΔcmH_2_0.ml^−1^s^−1^) or force (ΔmN) above baseline respectively. For small airway reactivity, raw data for airway lumen area in pixels were normalized to the initial area. Time course data was determined from individual frames collected at 2-second intervals. Average responses were obtained over the last minute of each perfusion condition.

All data were expressed as mean ± SEM, where each n represents a single mouse, or airway per mouse unless otherwise specified. Curves were compared by two-way Analysis of Variance (ANOVA), with Bonferroni post-hoc tests where appropriate. Measures of average potency (pEC_50_) and maximum responses obtained from individual curves fitted using Graph Pad Prism™ (version 5.0) were compared by unpaired t-tests. p<0.05 was accepted as being statistically significant.

## Results

### Chronic Allergen Challenge Induces Inflammation and Airway Remodelling

**Figure 1:**
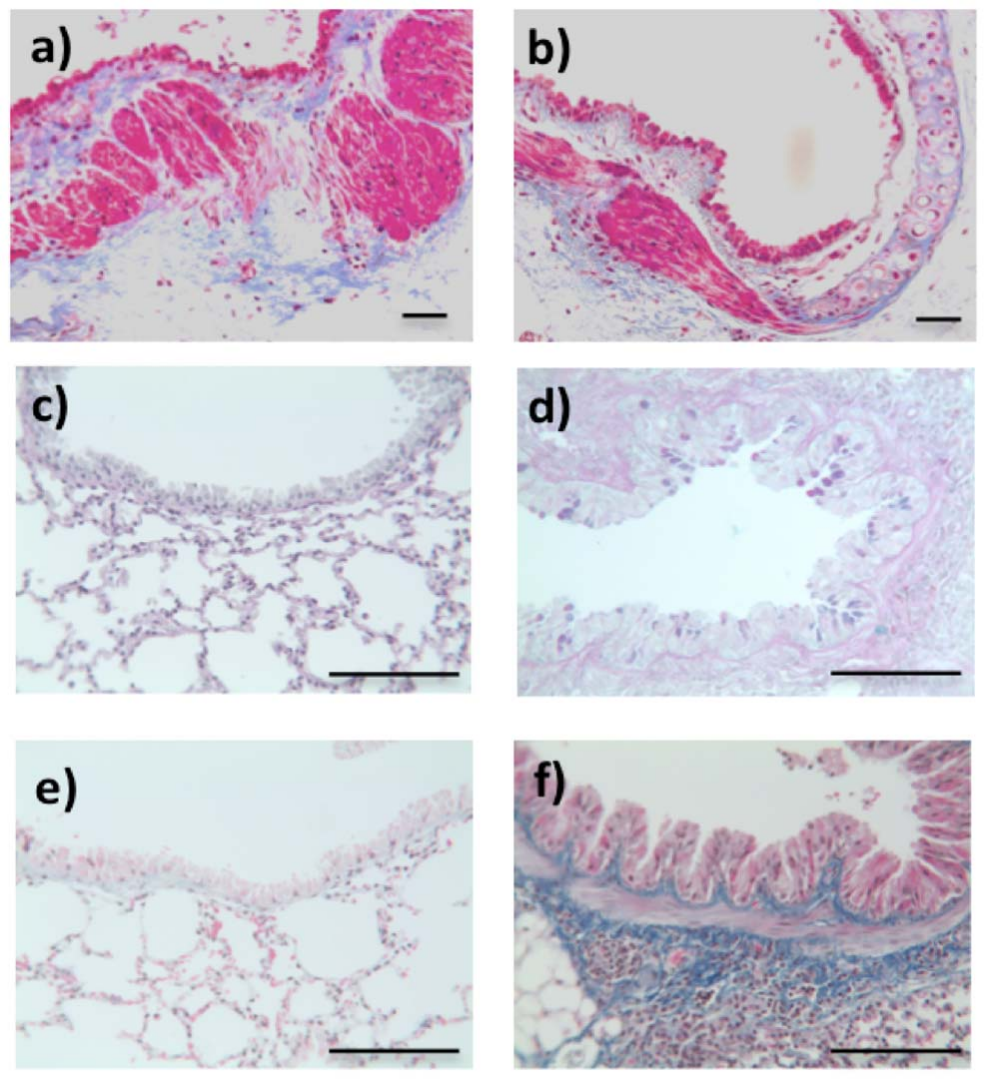
Changes to airway wall morphology with chronic allergen challenge. Representative sections from mouse trachea (panels a, b) and lung (panels c–f). Following chronic challenge with saline (a, c, e) or ovalbumin (b, d, f), sections were stained with Masson’s trichrome stain for collagen (a, b, e, f) or Alcian blue-periodic acid Schiff to assess epithelial changes (c, d). Bar = 100 µm.

This model of chronic AAD was initially validated, by demonstrating that total BAL cells were approximately 4-fold higher in OVA-challenged mice, with significant increases in all types of inflammatory cells, and a notably higher proportion of both eosinophils and lymphocytes (table 1). Histologic evaluation showed a marked increase in subepithelial collagen deposition following allergen challenge in small airways in lung sections, but not in trachea (fig. 1). There was also evidence of epithelial thickening and increased staining for mucin-containing goblet cells within lung sections from OVA-challenged mice (fig. 1).

**Table 1:** Bronchoalveolar Lavage Differential Cell Counts.

Cells×10^−3^	Total	Eosinophils	Neutrophils	Lymphocytes	Monocytes
**Saline**	95.0±50.8	0.5±0.2	0.8±0.1	10.0±2.0	83.7±18.8
**OVA**	385.0±29.4***	4.1±1.5***	6.1±2.7***	74.6±22.2***	300.2±37.6***

Bronchoalveolar lavage (BAL) fluid was collected from saline- and ovalbumin (OVA)-challenged mice (n = 6/group) and assessed for total cellularity. Differential cell counts were made on cytospin preparations from each BAL fluid sample based on counts of 300 cells. Results are expressed as mean ± SEM. Statistical analysis of log-transformed data was conducted using unpaired t-tests.

*** p<0.001 compared with saline.

### Allergen Challenge Alters Airway Responsiveness to MCh *in vivo* and *in vitro*

**Figure 2:**
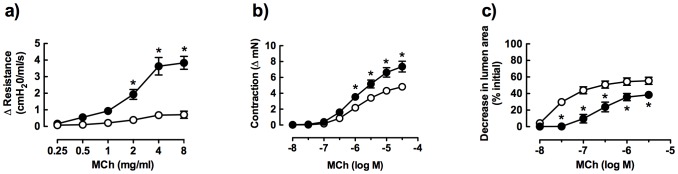
Comparison of airway reactivity to methacholine (MCh) in saline- and ovalbumin-challenged mice (open circles and closed circles respectively). a) *In vivo* responses, measuring change in airway resistance (saline, ovalbumin n = 12, 8 respectively), b) *in vitro* responses in trachea, measuring change in force (saline, ovalbumin n = 18, 21) and c) *in vitro* responses in lung slices, measuring % decrease in small airway lumen area (saline, ovalbumin n = 9, 7). Data is expressed as mean ± S.E.M. *p<0.05 compared with saline controls.

*In vivo* AHR to MCh was also clearly evident following OVA-challenge. MCh elicited a relatively modest increase in airway resistance in saline-challenged mice. The response to the highest dose of MCh tested was increased more than 4-fold after allergen challenge (two-way ANOVA, p<0.001; fig. 2a).

In tracheal rings from saline-challenged mice, MCh caused concentration-dependent contraction with an EC_50_ of 1.5±0.2 µM (fig. 2b, table 2). Although the potency of MCh was similar in saline- and OVA-challenged mice, the maximum increase in force was significantly higher in large airways following allergen challenge (unpaired t-test, p<0.05; table 2).

**Table 2:** Comparison Of Methacholine Potency And Maximum In Trachea And Small Airways In Vitro.

	Trachea		Small airway	
	Saline	OVA	Saline	OVA
**n**	18	21	9	7
**Maximum**	5.0±0.4	7.6±0.7*	55.5±5.0	44.5±3.5
	(ΔmN)		(% decrease in lumen area)	
**EC_50_**	1.5±0.2	1.5±0.3	23±5	360±102*
	(µM)		(nM)	

Airway reactivity to methacholine was assessed *in vitro* measuring increases in force in trachea and small airway narrowing in lung slices from saline- and ovalbumin (OVA)-challenged mice. Maximum responses and potency were obtained from fitted individual curves. Data is expressed as mean ± SEM.

* p<0.05 compared with saline, unpaired t-test.

Small airway reactivity was also assessed by measuring changes in airway lumen area in perfused lung slices. Notably, the potency of MCh was more than 50 times higher in small airways than in trachea from saline-challenged mice (table 2).

The decrease in lumen area in response to MCh was relatively reduced following OVA-challenge (unpaired t-test, p<0.05; fig. 2c), despite similar airway sizes in both groups (SAL: 310±17 µm internal diameter, n = 9; OVA: 340±25 µm, n = 7).

### Small Airway Contraction to MCh is Reduced Following Allergen Challenge

This effect of allergen challenge on responses to MCh in small airways was examined in more detail, showing its effects on lumen area and rate of contraction.

**Figure 3:**
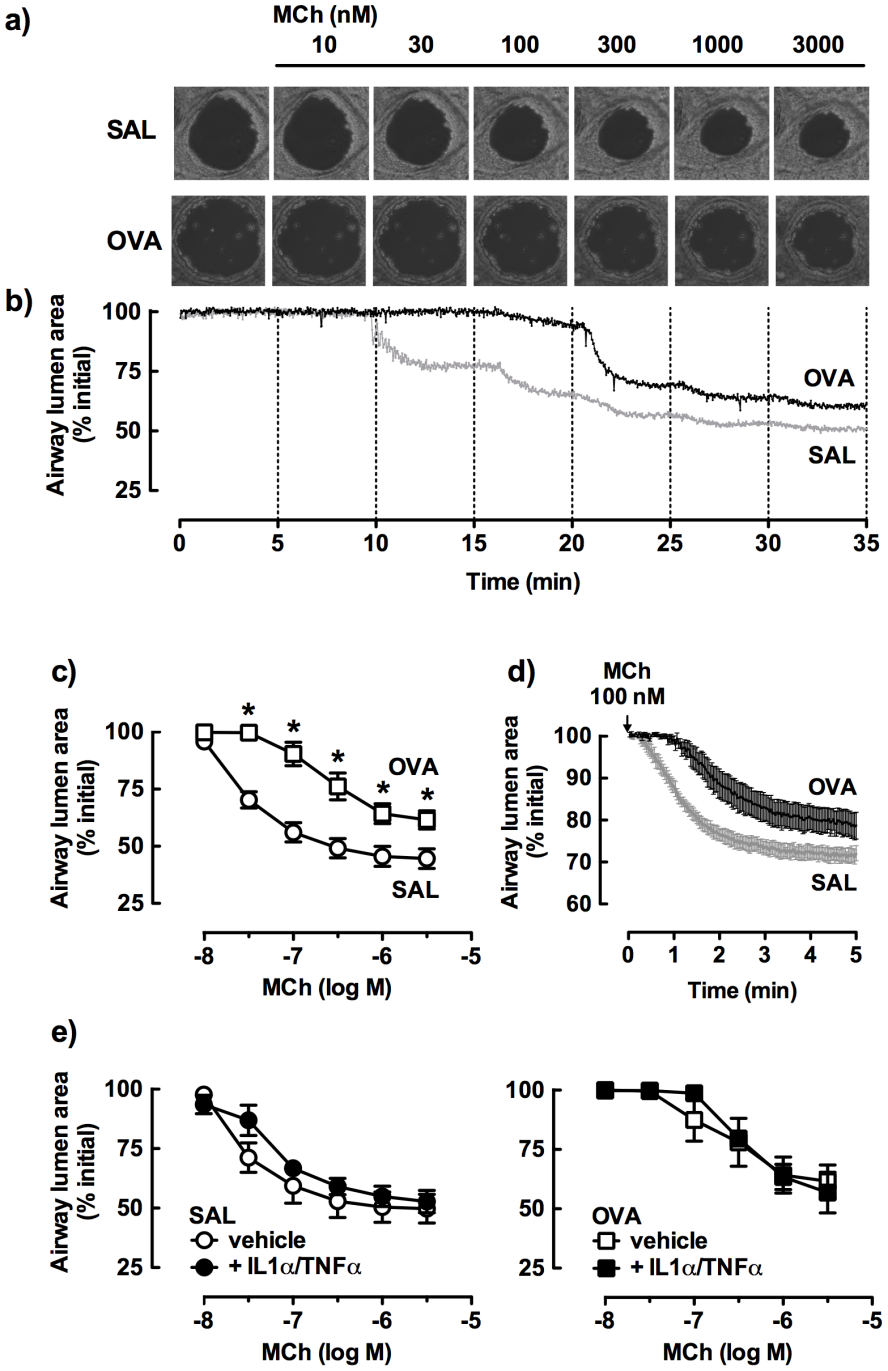
Comparison of small airway responses to methacholine (MCh) in lung slices from saline (SAL)- and ovalbumin (OVA)-challenged mice. a) Representative images of MCh-induced airway narrowing. b) Representative traces showing the time course of changes in small airway lumen area. c) Average changes in % initial airway lumen area (SAL, OVA n = 9, 7). d) Contraction to 100 nM MCh over 5 min (SAL, OVA n = 4, 5). e) Lung slices cultured in the absence (open symbols) or presence (closed symbols) of IL-1α (10 ng/ml) and TNFα (50 ng/ml) for 48 h (paired tissues, SAL or OVA n = 4, 4) Data is expressed as % initial airway lumen area (mean ± SEM). *p<0.05 compared with appropriate controls.

Comparison of sequential images of a small airway within a lung slice from a saline-or OVA-challenged mouse with increasing MCh concentrations showed threshold responses at 30 nM and 100 nM, respectively (fig. 3a). Representative traces recorded during MCh perfusion demonstrated the relatively reduced sensitivity of a single small airway from an OVA-challenged mouse relative to a saline control (fig. 3b), consistent with the group data showing a 15-fold loss in MCh potency (fig. 3c, table 2).

The time course of airway narrowing to a submaximal concentration of MCh (100 nM) during a single 5 min perfusion was also compared (fig. 3d). The relatively reduced contraction to MCh following OVA-challenge was associated with a delayed onset of ∼1 min and a reduced rate of contraction compared with the saline control.

Slices were incubated for 48 h with IL-1α and/or TNFα to assess the effect of *in vitro* exposure to inflammatory cytokines on small airway reactivity. Contractile responses to MCh were not altered by preincubation with these cytokines, added either alone (data not shown), or in combination (fig. 3e). In addition, the relatively lower tissue sensitivity to MCh following OVA-challenge was maintained.

### Possible Contribution of Endogenous PGE_2_ to MCh Responsiveness in Small Airways

Since it has been reported that PGE_2_ production in the lung may be increased after allergen challenge [22], we wanted to determine whether the continuous presence of PGE_2_ could inhibit the development of MCh-induced contraction in small airways. Using slices from allergen-naïve mice, which had the same sensitivity to MCh as airways from saline-challenged mice (EC_50_ values of 22±7 nM and 23±5 nM respectively), we showed that addition of 100 nM PGE_2_ almost completely reversed an established submaximal contraction to MCh (fig. S1a). Although continuous perfusion with 100 nM PGE_2_ was able to reduce the contraction of small airways to 10^−8^ M MCh, it was not able to oppose the response to higher concentrations of MCh, with no apparent loss of MCh potency or reduction in maximum contraction (fig. S1b). This was in marked contrast to lung slice data from saline- and OVA-challenged mice, where small airway contraction to perfusion with low MCh concentrations was similar, but MCh potency was markedly reduced after OVA challenge (fig. 2c, table 2).

### Reduced Small Airway Contraction to MCh Following Allergen Challenge is Not Associated with Reduced Calcium Sensitivity

**Figure 4:**
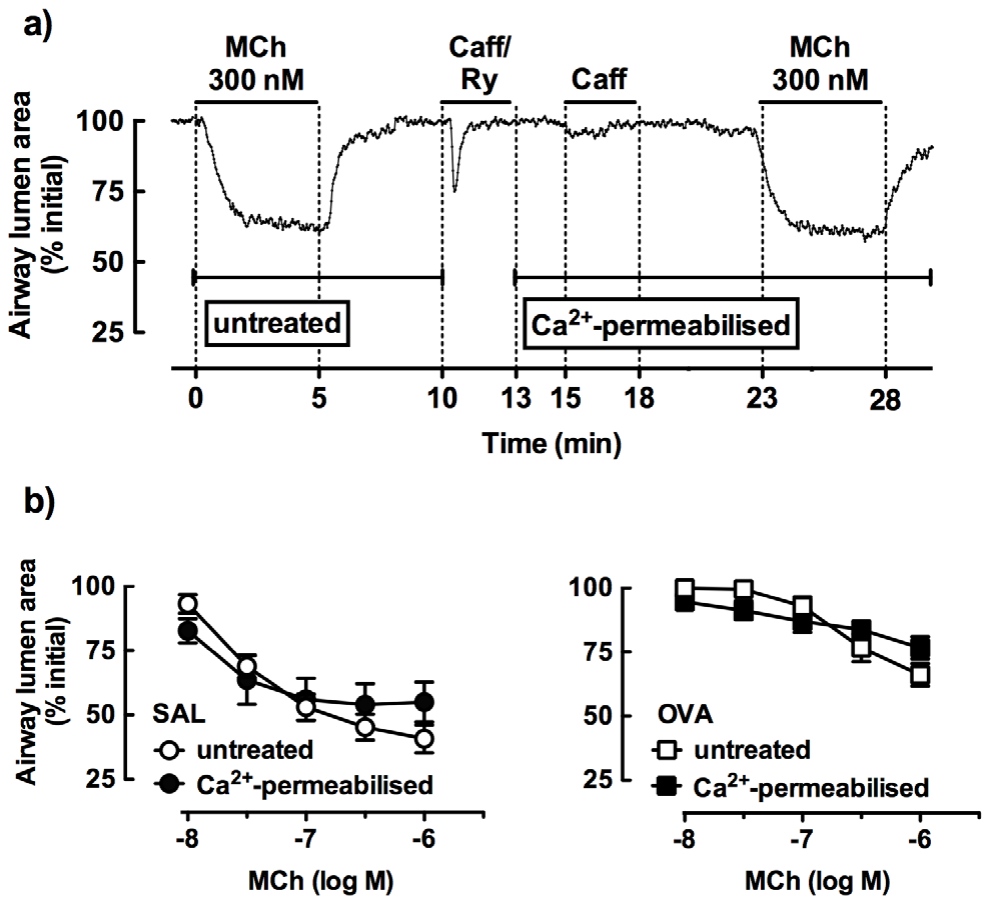
Comparison of small airway responses to methacholine (MCh) in lung slices from saline- and ovalbumin (OVA)-challenged mice under control and calcium-permeabilised conditions. Slices were treated with permeabilized to Ca^2+^ by simultaneous treatment with 20 mM caffeine and 50 µM ryanodine to open ryanodine receptors (RyR) in the sarcoplasmic reticulum of airway smooth muscle cells, depleting intracellular Ca^2+^ stores and clamping the intracellular Ca^2+^ concentration at extracellular levels. This abrogates subsequent caffeine contractions and MCh-induced Ca^2+^ oscillations, to permit the assessment of MCh responses due to RhoA/Rho kinase-mediated Ca^2+^ sensitisation alone [21]. a) Representative trace showing response to 300 nM MCh under control and calcium-permeabilised conditions. b) Concentration-response to MCh (paired tissues, SAL or OVA n = 5, 3) expressed as % initial airway lumen area (mean ± SEM).

To determine whether the reduced small airway reactivity to MCh after allergen challenge was associated with altered Ca^2+^ sensitivity, responses were compared in untreated and Ca^2+^-permeabilised lung slices from both saline- and OVA-challenged mice. A representative trace shows contraction to a single submaximal concentration of MCh, prior to treatment with caffeine/ryanodine that causes a transient airway contraction associated with release and depletion of intracellular Ca^2+^. Under these conditions, the subsequent response to caffeine is abolished, but MCh contraction is maintained at a similar level due to Ca^2+^ sensitivity (fig. 4a).

Full concentration-response curves showed that MCh-induced airway narrowing was similar in untreated airways and Ca^2+^-permeabilised airways in lung slices from saline-challenged mice (fig. 4b). The reduced responsiveness to MCh following OVA challenge was maintained after treatment of slices with caffeine/ryanodine (fig. 4b).

## Discussion

In the present study, AHR to MCh was established *in vivo* in a mouse model of chronic AAD and maintained in measurements of force in tracheal ring preparations *in vitro.* In contrast, small airway narrowing in response to MCh measured in perfused lung slices from the same model was reduced following allergen challenge. This unexpected loss of MCh potency was unaltered by *in vitro* exposure to inflammatory cytokines and was not associated with altered MCh-induced Ca^2+^ sensitivity.

AHR is a hallmark of asthma, associated with acute and chronic airway inflammation and associated tissue repair and airway remodelling. We initially demonstrated *in vivo* AHR to MCh in our chronic allergen challenge model, associated with inflammatory cell influx and airway wall remodeling within the lung, as previously reported [17]. Remodelling changes in lung sections were limited to epithelial thickening and increases in goblet cells and subepithelial collagen. This confirmed that allergen challenge can induce increased tracheal sensitivity to MCh, in the absence of ASM thickening.

Other potential contributors to AHR in inflamed, remodeled airways include changes in the intrinsic properties of ASM and/or its interactions with altered non-contractile elements within the airway wall and surrounding lung parenchyma [20]. We assessed these factors by integrating our *in vivo* assessment of changes in ASM reactivity with *in vitro* measurements in tracheal and small airway preparations.

Initially, we explored whether *in vitro* reactivity was differentially regulated in this model of chronic AAD with airway size. Increased contraction to MCh was evident in isolated tracheal rings after allergen challenge, demonstrating that continued *in vitro* exposure to an inflammatory environment was not required for the greater increase in force. This hyperresponsiveness has previously been attributed to altered expression of contractile proteins, such as an increased ratio of F-actin:G actin content [9], [10], changes in the composition of myosin isoforms and/or overexpression of myosin light chain kinase [23], rather than a change in contractile receptor expression [24].

Despite the clear contribution of small airways in the distal lung to asthma, studies of their reactivity *in vitro* after OVA challenge are limited to only a single report [14]. Using a similar chronic model of AAD to the present study, it was previously shown that the rate or extent of contraction to a single maximal concentration of ACh was not altered by allergen challenge [14]. In contrast, we have shown that small airways were hyporesponsive to a range of MCh concentrations after chronic OVA challenge. This was evident despite *in vivo* inflammation, remodelling and AHR and increased tracheal contraction to MCh in the same model.

The major focus of this study was the effect of chronic allergen challenge rather than acute treatment with cytokines on small airway reactivity. However, it has been proposed that sustained *ex vivo* exposure to an inflammatory environment may be required to maintain hyperresponsivess in small airways after allergen challenge [14]. We therefore assessed the effect of incubating lung slices with inflammatory cytokines IL-1α and TNFα, alone or in combination for 48 h, under conditions that induced AHR *in vitro* to MCh in trachea from allergen naive mice [15]. However, small airway contraction to MCh in lung slices from either saline- or OVA-challenged mice was not altered by prior incubation with these cytokines. A previous report has demonstrated that a cytokine mix containing IL-1 and TNFα, at lower concentrations than tested in the present study, directly induced acute contraction of small airways over 4 h [25], but MCh sensitivity was not measured in this setting.

Recent studies have implicated diverse cytokines including IL-13 [26] and IL-17 [27] in the acute regulation of small airway reactivity and increased sensitivity to contractile agonists. In future studies, we propose to assess whether the effects of OVA challenge on small airway reactivity to MCh seen in this study can be modulated by acute treatment with alternative disease-relevant Th2 cytokines such as IL-4, IL-5 or IL-13 and/or by varying the duration of exposure *in vitro*.

Previous studies suggest that chronic allergen challenge induces increased production of the endogenous dilator PGE_2_ [22]. However, this was not measured in the current study and assessment of both tracheal and small airway reactivity were performed in the absence of indomethacin. It is possible therefore that tracheal hyperresponsiveness to MCh following OVA challenge could have been underestimated. Under the static organ bath conditions used, any increase in tracheal PGE_2_ production and its accumulation could partially oppose MCh-mediated contraction.

Increased production of PGE_2_ by small airways after OVA challenge could provide a mechanism contributing to the decreased contraction to MCh observed in lung slices. To address this, we assessed whether continuous perfusion of small airways from allergen-naïve mice with PGE_2_ (to simulate any increase in endogenous PGE_2_ production after allergen challenge) was able to inhibit the development of MCh-induced reductions in small airway lumen area. Although neither MCh potency or maximal contraction appeared to be reduced under these conditions, the contribution of increased PGE_2_ production by small airways to MCh hyporesponsiveness after OVA challenge cannot be completely excluded. This should be tested under conditions where the removal of endogenous mediators produced by perfusion of the tissues is minimised, to assess the impact of PGE_2_ and other prostaglandins that may influence airway reactivity. Nevertheless, the current study suggests that alternative or additional mechanisms underly the hyporesponsiveness to MCh hyporesponsiveness as measured in perfused lung slices after OVA challenge.

A possible mechanistic explanation for the reduced MCh potency in small airways from OVA-challenged mice is reductions in ASM calcium signalling and/or sensitivity. Airway contraction in response to agonists such as ACh and 5-HT is dependent on increased [Ca^2+^]_i_, provided by Ca^2+^ oscillations mediated by repetitive Ca^2+^ release from internal stores [19]. Although impairment of MCh-induced increases in rate and frequency of Ca^2+^ oscillations in ASM after OVA challenge may provide a potential mechanism for the slower rate of MCh-induced contraction of small airways, we have yet to address this possibility.

Although this increase in [Ca^2+^]_i_ initiates contraction, RhoA/Rho kinase-mediated Ca^2+^ sensitisation is also important for sustained contraction [21]. Augmented Ca^2+^ sensitisation has previously been associated with hyperresponsiveness to ACh in α-toxin-permeabilised epithelial-denuded mouse bronchial strips [24]. In the current study, mouse lung slices were permeabilised with caffeine/ryanodine, to lock RyR in an open state leading to depletion of the ASM intracellular Ca^2+^ stores. The subsequent increase in Ca^2+^ influx via store-operated channels across the plasma membrane clamps the intracellular Ca^2+^ concentration of ASM at extracellular levels and abolishes subsequent MCh-induced Ca^2+^ oscillations [21]. Using Ca^2+^-permeabilised lung slices, we confirmed that MCh potency and efficacy, now due to RhoA/Rho kinase-mediated Ca^2+^ sensitisation alone, were unchanged in comparison with untreated slices [21]. In addition, the small airway hyporesponsiveness to MCh was maintained after caffeine/ryanodine treatment of lung slices obtained after chronic OVA challenge. This suggests that the mechanism underlying this reduced reactivity is unlikely to be associated with an allergen-induced impairment of Ca^2+^ sensitivity.

Our *in vitro* findings demonstrated regional differences in contractility after OVA challenge, with decreased and increased sensitivity to MCh in small and large airways respectively. This is in contradistinction to a previous report, in which AHR to both ACh and ET-1 was evident in measurements of increased force development in bronchial but not tracheal rings after allergen challenge [24]. This was attributed to greater inflammation rather than increased receptor expression levels in bronchi [24]. Given these combined findings, future studies should compare relative inflammation down to the level of the small airways assessed here, as well as characterising any changes in responsiveness, receptor expression and/or subsequent signalling for diverse contractile agonists such as serotonin to determine if the observed small airway hyporesponsiveness is specific to MCh.

In the current study, we have used different techniques to assess MCh reactivity in trachea and small airways. We propose that the increased development of contractile force to MCh in isolated tracheal rings demonstrates the influence of allergen challenge on intrinsic ASM contraction, unopposed by any recoil forces. Since measurements of small airway lumen area *in situ* within lung slices preserve the interdependency of ASM with the surrounding tissue, we propose that our findings of reduced MCh contraction incorporate the additional influence of allergen-induced structural changes on the airway and/or its interactions with surrounding parenchyma that are not detected when measuring responses in tracheal rings *in vitro*.

In comparison with small airways from saline-challenged mice matched for internal diameter, the epithelial thickening seen following OVA challenge relative to could have resulted in increased internal and external diameters of the smooth muscle layer and a relatively increased area. However, even if this were the case, the remodelled airways exhibited hyporesponsiveness to MCh rather than an increased contractile response.

One of the prominent features of remodelling seen in our model of AAD was marked subepithelial fibrosis in the small airways. We speculate that this supports the possibility that the stiffening of the airway and the surrounding parenchyma following allergen challenge may oppose ASM contractile forces. It has previously been demonstrated that increasing parenchymal stiffness minimizes airway narrowing *in vivo* [28]. In contrast, reducing airway-parenchymal interactions and airway collagen by treatment of human bronchial strips with collagenase has been shown to increase responsiveness of ASM to contractile stimuli [29]. In relation to small airways in lung slices, an increased velocity of ASM shortening associated with decreased mechanical load was evident following treatment with elastase and collagenase [30]. The current finding of reduced small airway reactivity to MCh is consistent with a previous study in Brown-Norway rats in which AHR was evident following short-term allergen exposure accompanied by airway wall thickening and inflammation, but could not be detected when long-term OVA challenge significantly increased submucosal collagen deposition [31]. To confirm the role of fibrosis in opposing small airway contraction, it would be of interest to assess whether collagenase treatment of lung slices from allergen-challenged mice would abrogate the reduced and slower contraction to MCh we have seen in the small airways.

In summary, it is possible that our contrasting findings in isolated tracheal rings and small airways reflect the differential influence of allergen challenge on airway inflammation and remodelling and their conferred effects on ASM reactivity depending on airway size. It is also feasible that that the assessment of small airway responses *in situ* within lung slices has revealed the potential influence of small airway fibrosis and altered parenchymal interactions after allergen challenge to reduce the rate and extent of airway narrowing and inflammation-induced AHR to MCh in the distal lung.

## Supporting Information

Figure S1 **Effect of PGE_2_ on small airway contraction to methacholine (MCh) in mouse lung slices.** a) Response to 300 nM MCh before and during perfusion with 100 nM PGE_2_ (n = 3). b) Response to MCh in the absence (open circles, n = 4) or presence (closed circles, n = 4) of 100 nM PGE_2_. Data is expressed as % initial airway lumen area (mean ± SEM). *p<0.05 compared with control.
(TIF)
