# Supplementary figures and images for: Differential Effects of Allergen Challenge on Large and Small Airway Reactivity in Mice
Source: PLoS One. 2013 Sep 6;8(9):e74101. doi: 10.1371/journal.pone.0074101 (PMC3765301; doi:10.1371/journal.pone.0074101)

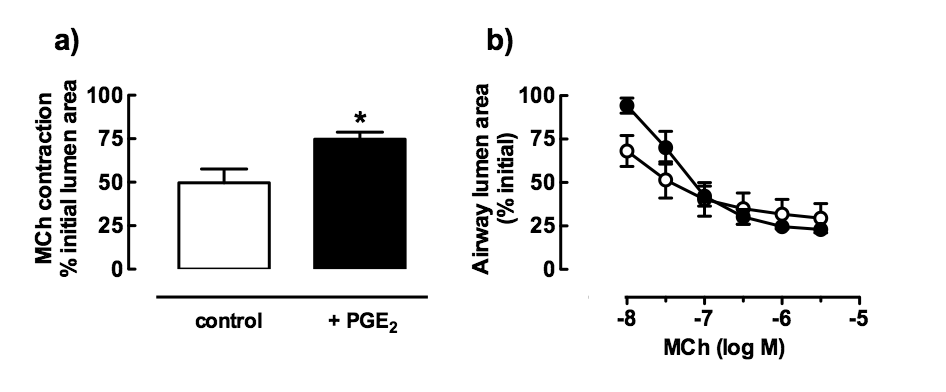

Supplement: Figure S1 — Effect of PGE2 on small airway contraction to methacholine (MCh) in mouse lung slices. a) Response to 300 nM MCh before and during perfusion with 100 nM PGE2 (n = 3). b) Response to MCh in the absence (open circles, n = 4) or presence (closed circles, n = 4) of 100 nM PGE2. Data is expressed as % initial airway lumen area (mean ± SEM). *p<0.05 compared with control. (TIF) [file pone.0074101.s001.tif]
